# Comparison of the effect of intra-cuff normal saline, dexamethasone or ketamine for prevention of postoperative sore throat: a randomized controlled trial

**DOI:** 10.1016/j.bjane.2025.844651

**Published:** 2025-06-13

**Authors:** Arun Mukesh, Ankur Sharma, Priyabrat Karan, Darshna Rathod, Shilpa Goyal, Kamlesh Kumari, Manbir Kaur, Tanvi Meshram, Pradeep Bhatia

**Affiliations:** a All India Institute of Medical Sciences (AIIMS), Department of Anesthesiology & Critical Care, Jodhpur, India; b All India Institute of Medical Sciences (AIIMS), Department of Trauma & Emergency (Anesthesiology & Critical Care), Jodhpur, India

**Keywords:** Cough, Dexamethasone, Hoarseness, Ketamine, Pain, Postoperative

## Abstract

**Background:**

Postoperative Sore Throat (POST) may result in patient dissatisfaction and distress, which could possibly delay discharge. Various pharmacological and non-pharmacological approaches have been explored, yet effective techniques remain elusive. This research evaluates the impact of intra-cuff Dexamethasone, Ketamine, and normal saline on alleviating POST symptoms.

**Methods:**

In this randomized controlled trial, 405 adult patients aged 18‒60 years undergoing short pelvic laparoscopic surgeries under general anesthesia for 1‒3 h requiring endotracheal intubation were enrolled. Patients were randomized into Group N (intra-cuff normal saline), Group D (intra-cuff Dexamethasone), and Group K (intra-cuff Ketamine). The primary outcome of this study was the incidence and severity of POST at 2, 6, 12, and 24 hours after extubation. Secondary outcomes were the incidence and severity of postoperative hoarseness of voice and postoperative cough at various time intervals.

**Results:**

There were more patients in Group D without symptoms of POST (92.59 %) than in Group K (74.07 %) and Group N (67.41 %) (*p* < 0.0001) at 2 h. Similarly, more patients had no symptoms of postoperative hoarseness of voice (93.33 %) and postoperative cough (93.33 %) in Group D at 2 h. Furthermore, Group D consistently exhibited the lowest incidence of POST, postoperative hoarseness of voice, and postoperative cough at various time intervals.

**Conclusions:**

Intra-cuff Dexamethasone appears to be a favourable intervention for symptom alleviation of POST, postoperative hoarseness of voice, and postoperative cough during the early postoperative period.

**Clinical Trial Registry Number:**

CTRI/2022/08/044,664.

## Introduction

Postoperative Sore Throat (POST) is a usual complication after general anesthesia with endotracheal tube intubation. The prevalence of sore throat after tracheal intubation in the postoperative phase ranges from 21 % to 65 %.^1^ Despite a self-limiting condition, it may postpone discharge after day-care procedures. It eventually leads to unpleasant memories and dissatisfaction in patients in the postoperative period.^2^ POST is known to be an aseptic inflammatory process due to localized trauma to the mucosa during airway manipulation.

POST may be prevented using a variety of pharmacological and non-pharmacological strategies. Among non-pharmacological methods, the use of small-size tracheal tubes, supra-glottic devices, meticulous airway instrumentation, gentle suction of the oropharynx, application of water-soluble jelly over the tracheal tube, and low intra-cuff pressure has been studied in the literature.^3^ Among pharmacological agents, Dexamethasone, lignocaine, and magnesium sulfate have been used in various studies.^4^

Studies have shown that a peripherally administered N-Methyl-d-Aspartate (NMDA) receptor antagonist like Ketamine has demonstrated anti-nociceptive and anti-inflammatory effects. Dexamethasone is a potent corticosteroid with anti-inflammatory action. Because of its ability to modulate tissue edema and discomfort, it has been used to treat sore throats caused by tracheal irritation.^5^, ^6^, ^7^

The present study aimed to compare intra-cuff normal saline, Dexamethasone, and Ketamine to reduce Postoperative Sore Throat (POST) in patients undergoing surgery under general anesthesia with endotracheal intubation. We hypothesized that there is no difference in the incidence and severity of POST in the three groups.

## Methods

### Study settings

The present study was conducted within the operation theatres of an academic tertiary hospital. Following clearance from the ethics committee (AIIMS/IEC/2022/3906), this trial was subsequently registered with the Clinical Trials Registry of India (CTRI/2022/08/044,664) (https://ctri.nic.in/Clinicaltrials/rmaindet.php?trialid=67,337&EncHid=49,341.90645&modid=1&compid=19).

### Patients

Adult patients aged between 18 and 60 years of either sex with American Society of Anesthesiologists (ASA) physical status 1‒2 with Mallampati grades I or II undergoing short pelvic laparoscopic surgeries with a duration greater than 1 hour and lasting less than 3 h under general anesthesia in supine position requiring endotracheal intubation were recruited in this study. The exclusion criteria were patient’s refusal to participate, history of pre-operative sore throat, smoker, oral and nasal surgeries, upper respiratory tract infection, pregnant females, patients with chronic obstructive pulmonary disease, known allergies to study drugs, anticipated airway instrumentation difficulty with Mallampati grades III/IV, and patients who required more than one attempt for intubation.

### Interventions

Patients were randomized into Group N (intra-cuff normal saline), Group D (intra-cuff Dexamethasone), and Group K (intra-cuff Ketamine) by a web‑based randomization program (www.randomizer.org), and the randomization sequence was kept inside serially numbered opaque‑sealed envelope. Sealed envelopes were opened to reveal allocation before inducing the patient for general anesthesia. Both the patients and assessors were blinded to group allocation.

A standardized protocol was followed to administer general anesthesia. All patients had preoxygenation for 3 min with 100 % oxygen prior to the administration of anesthesia. Induction in all patients was accomplished with Intravenous (IV) fentanyl at 1.5–2 μg.kg^−1^, IV propofol at 2–2.5 mg.kg^−1^, IV atracurium at a dosage of 0.5 mg.kg^−1^, and 100 % oxygen. After mask ventilation for 3 min, participants were intubated by a swift and gentle laryngoscopy that lasted no more than 15 s, using a low-pressure, high-volume, cuffed polyvinyl chloride Endotracheal Tube (ETT). In male patients, ETT with an internal diameter of 8 mm was used, whereas, in female patients, ETT with a 7 mm internal diameter was utilized. Endotracheal intubation was performed by two anesthesiologists (A.M. and A.S.), who had more than five years of experience and was verified by bilateral air entry upon auscultation and a consistent end-tidal capnographic waveform.

Two other anesthesiologists (P.K. and D.R.) filled the study drug in the endotracheal tube cuff with the minimum volume required to prevent an audible leak. Patients in the N, D, and K groups received intra-cuff normal saline, 0.1 mg.kg^−1^ of Dexamethasone, and 0.5 mg.kg^−1^ of Ketamine, respectively. In groups D and K, the estimated doses of Dexamethasone and Ketamine were first administered to the endotracheal tube cuff, followed by the desired amount of saline. Anesthesia was maintained using a mixture of oxygen and air (1:2) containing 1 %–1.2 % isoflurane (end-tidal, 0.7 to 1 MAC). After surgery, ondansetron 0.1 mg.kg^−1^ was administered intravenously, and residual muscle paralysis was reversed with neostigmine 0.05 to 0.07 mg.kg^−1^ and glycopyrrolate 10 μg.kg^−1^ IV. In all patients, following mild oropharyngeal suctioning, extubation was carried out after completion of surgery. All patients were administered 1 g of paracetamol intravenously at an 8-hour interval. Patients were assessed and graded for POST, hoarseness of voice, and postoperative cough at 2, 6, 12, and 24 hours postoperatively by independent anesthesiologists who were not part of this study using a scoring chart (Suppl. File 1).

The primary outcome of this research was the occurrence and intensity of POST at 2, 6, 12, and 24 hours post-extubation. Secondary outcomes were the incidence and severity of postoperative hoarseness of voice and postoperative cough at various time intervals.

### Sample size

Rajan S et al. have reported 24-hour POST in 36.7 % of the saline group and 0 % in the Dexamethasone group.^8^ To estimate a 50 % decrease in the incidence of post-op sore throat, we estimated a sample size of 135 per group at 95 % CI, 80 % power adjusted for three groups, and 10 % contingency.

### Data analysis

The data was entered into a Microsoft Excel spreadsheet, and the final analysis was performed with the Statistical Package for Social Sciences (SPSS) software, IBM, Chicago, USA, version 25.0. The categorical variables were reported as numbers and percentages. The quantitative data were provided as means ± SD and median, along with the 25^th^ and 75^th^ percentiles (interquartile range). The comparison of the quantitative variables was analyzed using the Analysis of Variance (ANOVA) test with Bonferroni correction. The comparison of the qualitative variables was analyzed using the Chi-Square test and Fisher's exact test. For statistical significance, a *p*-value of less than 0.05 was considered statistically significant. The analysis was performed after the sample size was completed and relevant follow-up was performed.

## Results

**Figure 1:**
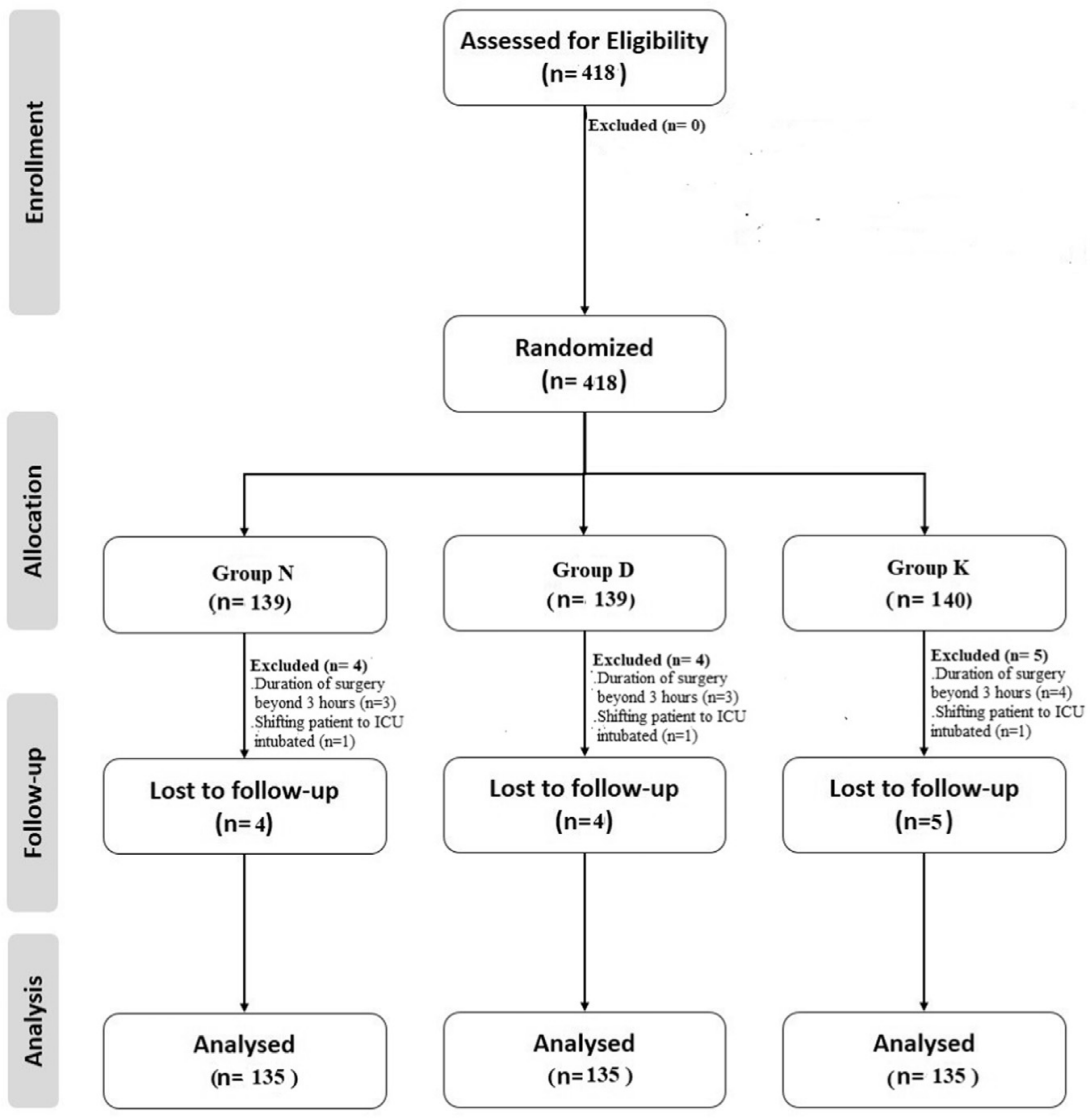
Consort diagram.

Initially, 418 patients were enrolled in the trial; however, 13 were excluded after randomization due to surgery lasting more than 3 h or the patient being kept under mechanical ventilation after the procedure (4 in the N group, 4 in the D group, and 5 in the K group). Following the per-protocol analysis, a total of 405 patients were included in the final analysis and allocated to three study groups, with 135 patients in each group (Figure 1). The excluded 13 patients were managed at the discretion of the anesthesiologist posted in the operation theatre. All three study groups had similar demographic characteristics (Table 1).

**Table 1:** Comparison of various characteristics between the groups.

Parameters		Group N (*n* = 135)	Group D (*n* = 135)	Group K (*n* = 135)	Total	*p*-value
Age (years)		42.43 ± 11.08	43.28 ± 12.2	43.98 ± 10.76	43.23 ± 11.35	0.534
Gender	M	71 (52.59 %)	69 (51.11 %)	70 (51.85 %)	210 (51.85 %)	0.971
	F	64 (47.41 %)	66 (48.89 %)	65 (48.15 %)	195 (48.15 %)	
Height (cm)		168.56 ± 6.11	170.56 ± 7.02	165.34 ± 9.05	168.15 ± 7.78	0.074
Weight (Kg)		66.24 ± 16.02	67.27 ± 13.09	65.43 ± 12.15	66.31 ± 13.84	0.550

M, Male, F, Female.

Group N, D, and K: Group Normal saline, Dexamethasone, and Ketamine, respectively.

**Figure 2:**
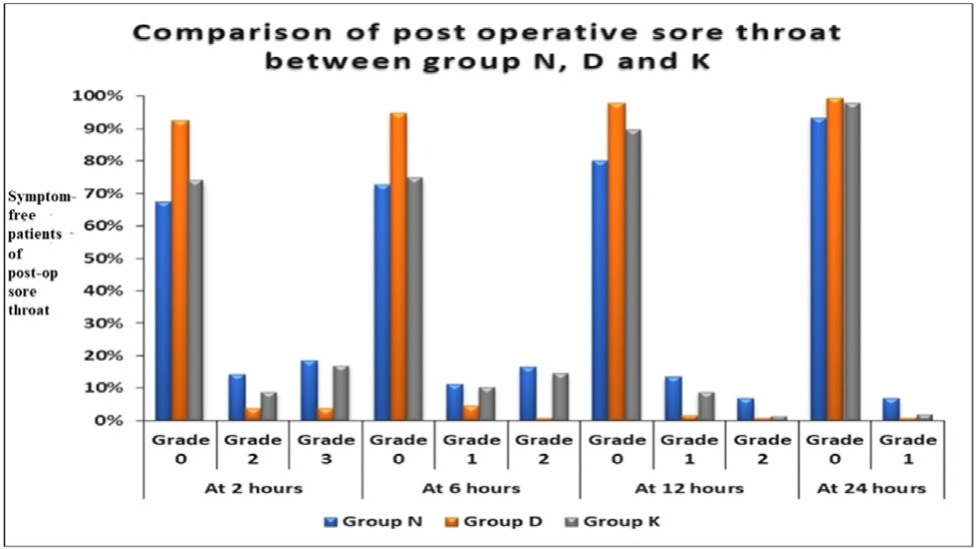
Comparison of post-operative sore throat among groups N, D and K at various intervals.

In the following two hours, in Group D, 92.59 % of patients had no symptoms (Grade 0) for POST, followed by Group K (74.07 %) and Group N (67.41 %). There was a significant difference between Group N and Group D (*p* < 0.0001) and between Group D and Group K (*p* = 0.0002) at 2 h. However, there was no significant difference between Group N and Group K (*p* = 0.352) at this time. After 6 h, similar trends were observed, with Group D having no symptoms of POST (Grade 0) in 94.81 % of patients, followed by Group K (74.81 %) and Group N (72.59 %). At 12 h, in Group D, 97.78 % had no symptoms of POST, followed by Group K (89.63 %) and Group N (80 %). At 24 h, in Group D, 99.26 % had no POST symptoms (Grade 0), followed by Group K (97.78 %) and Group N (93.33 %). At 24 h, there was a substantial difference between groups N and D (*p* = 0.019). In contrast, no significant differences were observed between Group N and Group K (*p* = 0.137) or between Group D and Group K (*p* = 0.622) at this time. There were no patients in Grade 3 POST in any of the groups (Figure 2, Table 2).

**Table 2:** Comparison of post-operative sore throat between groups N, D, and K.

Symptom-free patients of post-operative sore throat	Group N (*n* = 135)	Group D (*n* = 135)	Group K (*n* = 135)	*p*-value (effect size)
**At 2 h**				
Grade 0	91 (67.41 %) [59.11, 74.74]	125 (92.59 %) (86.90, 95.93)	100 (74.07 %) (66.09, 80.73)	< 0.0001^b^ N vs. D: < 0.0001^b^ (0.315) N vs. K: 0.352^b^ (0.087) D vs. K: 0.0002^b^ (0.252)^b^
Grade 2	19 (14.07 %) [9.20, 20.94]	5 (3.70 %) (1.59, 8.38)	12 (8.89 %) (5.16, 14.89)	
Grade 3	25 (18.52 %) [12.87, 25.91]	5 (3.70 %) [1.59, 8.38]	23 (17.04 %) [11.63, 24.27]	
**At 6 h**				
Grade 0	98 (72.59 %) [64.52, 79.41]	128 (94.81 %) [89.68, 97.47]	101 (74.81 %) [66.88, 81.38]	< 0.0001^b^ N vs. D: < 0.0001^b^ (0.316) N vs. K: 0.916^b^ (0.025) D vs. K: < 0.0001^b^ (0.295)^b^
Grade 1	15 (11.11 %) [6.85, 17.52]	6 (4.44 %) [2.05, 9.36]	14 (10.37 %) [6.28, 16.66]	
Grade 2	22 (16.30 %) [11.02, 23.44]	1 (0.74 %) [0.13, 4.08]	20 (14.81 %) [9.80, 21.78]	
**At 12 h**				
Grade 0	108 (80 %) [72.46, 85.88]	132 (97.78 %) [93.67, 99.24]	121 (89.63 %) [83.34, 93.72]	< 0.0001^a^ N vs. D: < 0.0001^b^ (0.282) N vs. K: 0.041^b^ (0.153) D vs. K:0.007^a^ (0.171)
Grade 1	18 (13.33 %) [8.60, 20.09]	2 (1.48 %) [0.41, 5.24]	12 (8.89 %) [5.16, 14.89]	
Grade 2	9 (6.67 %) [3.55, 12.18]	1 (0.74 %) [0.13, 4.08]	2 (1.48 %) [0.41, 5.24]	
**At 24 h**				
Grade 0	126 (93.33 %) [87.82, 96.45]	134 (99.26 %) [95.92, 99.87]	132 (97.78 %) [93.67, 99.24]	0.027^a^ N vs. D: 0.019^a^ (0.137) N vs. K: 0.137^a^ (0.089) D vs. K: 0.622^a^ (0.030)
Grade 1	9 (6.67 %) [3.55, 12.18]	1 (0.74 %) [0.13, 4.08]	3 (2.22 %) [0.76, 6.33]	

a Fisher's exact test.

b Chi-Square test. Values are in n (%) and 95 % Confidence Interval (95 % CI) of percentage. Group N, D, and K: Group Normal saline, Dexamethasone, and Ketamine, respectively.

For postoperative hoarseness, at 2 h, Group D had Grade 0 hoarseness (93.33 %), followed by Group K (82.96 %) and Group N (77.78 %). There was a significant difference between Group N and Group D (*p* = 0.001) and between Group D and Group K (*p* = 0.004). At 6 h, Group D showed Grade 0 hoarseness (99.26 %), followed by Group K (91.11 %) and Group N (82.96 %). At 12 h, Group D had Grade 0 hoarseness (100 %), followed by Group K (96.30 %) and Group N (90.37 %). At 24 h, no significant differences were observed between any of the groups at this time point (Table 3).

**Table 3:** Comparison of post-operative hoarseness between groups N, D and K.

Symptom-free patients of postoperative hoarseness	Group N (*n* = 135)	Group D (*n* = 135)	Group K (*n* = 135)	*p*-value (effect size)
**At 2 h**				
Grade 0	105 (77.78 %) [70.76, 84.79]	126 (93.33 %) (89.13, 97.54)	112 (82.96 %) [76.62, 89.30]	0.002^a^ N vs D: 0.001^a^ (0.235) N vs K: 0.372^a^ (0.109) D vs K: 0.004^a^ (0.212)
Grade 1	12 (8.89 %) [4.09, 13.69]	6 (4.44 %) [0.97, 7.92]	5 (3.70 %) [0.52, 6.89]	
Grade 2	14 (10.37 %) [5.23, 15.51]	3 (2.22 %) [0.00, 4.71]	13 (9.63 %) [4.65, 14.61]	
Grade 3	4 (2.96 %) [0.10, 5.82]	0 (0 %) [0.00, 0.00]	5 (3.70 %) [0.52, 6.89]	
**At 6 h**				
Grade 0	112 (82.96 %) [76.62, 89.30]	134 (99.26 %) [97.81, 100.00]	123 (91.11 %) [86.31, 95.91]	0.0001^b^ N vs. D: < 0.0001^a^ (0.286) N vs. K: 0.116^b^ (0.126) D vs. K: 0.003^a^ (0.192)^a^
Grade 1	14 (10.37 %) [5.23, 15.51]	1 (0.74 %) [0.00, 2.19]	6 (4.44 %) [0.97, 7.92]	
Grade 2	9 (6.67 %) [2.46, 10.87]	0 (0 %) [0.00, 0.00]	6 (4.44 %) [0.97, 7.92]	
**At 12 h**				
Grade 0	122 (90.37 %) [85.39, 95.35]	135 (100 %) [100.00, 100.00]	130 (96.30 %) [93.11, 99.48]	< 0.0001^a^ N vs. D: 0.0002^a^ (N.C.) N vs. K: 0.01^a^ (0.177) D vs. K: 0.06^a^ (0.137)
Grade 1	13 (9.63 %) [4.65, 14.61]	0 (0 %) [0.00, 0.00]	3 (2.22 %) [0.00, 4.71]	
Grade 2	0 (0 %) [0.00, 0.00]	0 (0 %) [0.00, 0.00]	2 (1.48 %) [0.00, 3.52]	
**At 24 h**				
Grade 0	133 (98.52 %) [96.48, 100.00]	135 (100 %) [100.00, 100.00]	131 (97.04 %) [94.18, 99.90]	0.214^a^ N vs. D: 0.498^a^ (N.C) N vs. K: 0.684^a^ (0.067) D vs. K: 0.122^a^ (0.122)
Grade 1	2 (1.48 %) [0.00, 3.52]	0 (0 %) [0.00, 0.00]	3 (2.22 %) [0.00, 4.71]	
Grade 2	0 (0 %) [0.00, 0.00]	0 (0 %) [0.00, 0.00]	1 (0.74 %) [0.00, 2.19]	

a Fisher's exact test.

b Chi-square test. N.C., Not Computable. Values are in n (%) and 95 % Confidence Interval (95 % CI) of percentage. Group N, D, and K: Group Normal saline, Dexamethasone, and Ketamine, respectively.

For postoperative cough, at 2 h, Group D had Grade 0 cough (93.33 %), followed by Group N (76.30 %) and Group K (74.81 %). There was a significant difference between Group N and Group D (*p* < 0.0001) and between Group D and Group K (*p* < 0.0001), but not between Group N and Group K (*p* = 0.558) at this time. At 6 h, Group D had Grade 0 cough (97.78 %), followed by Group K (82.22 %) and Group N (74.07 %). At 12 h, Group D had Grade 0 cough (99.26 %), followed by Group K (95.56 %) and Group N (82.96 %). At 24 h, Group D had Grade 0 cough (100 %), followed by Group K (99.26 %) and Group N (94.07 %). There was a significant difference between Group N and Group D (*p* = 0.007), between Group N and Group K (*p* = 0.036), but not between Group D and Group K (*p* = 1) (Table 4). The absolute risk reductions and Number Needed to Treat (NNT) for clinically relevant outcomes are represented in Suppl. Tables 1, 2, and 3.

**Table 4:** Comparison of postoperative cough between groups N, D, and K.

Symptom-free patients of postoperative cough	Group N (*n* = 135)	Group D (*n* = 135)	Group K (*n* = 135)	*p*-value (effect size)
**At 2 h**				
Grade 0	103 (76.30 %) [69.12, 83.47]	126 (93.33 %) [89.13, 97.54]	101 (74.81 %) [67.49, 82.14]	0.0005^b^ N vs. D: < 0.0001^a^ (0.269) N vs. K: 0.558^b^ (0.087) D vs. K: < 0.0001^a^ (0.268)
Grade 1	4 (2.96 %) [0.10, 5.82]	4 (2.96 %) [0.10, 5.82]	7 (5.19 %) [1.44, 8.93]	
Grade 2	18 (13.33 %) [7.60, 19.07]	5 (3.70 %) [0.52, 6.89]	21 (15.56 %) [9.44, 21.67]	
Grade 3	10 (7.41 %) [2.99, 11.83]	0 (0 %) [0.00, 0.00]	6 (4.44 %) [0.97, 7.92]	
**At 6 h**				
Grade 0	100 (74.07 %) [66.68, 81.47]	132 (97.78 %) [95.29, 100.26]	111 (82.22 %) [75.77, 88.67]	< 0.0001^a^ N vs. D: < 0.0001^a^ (0.344) N vs. K: 0.031^a^ (0.171) D vs. K: < 0.0001^a^ (N.C)
Grade 1	19 (14.07 %) [8.21, 19.94]	3 (2.22 %) [−0.26, 4.71]	20 (14.81 %) [8.82, 20.81]	
Grade 2	15 (11.11 %) [5.81, 16.41]	0 (0 %) [0.00, 0.00]	4 (2.96 %) [0.10, 5.82]	
Grade 3	1 (0.74 %) [−0.71, 2.19]	0 (0 %) [0.00, 0.00]	0 (0 %) [0.00, 0.00]	
**At 12 h**				
Grade 0	112 (82.96 %) [76.62, 89.30]	134 (99.26 %) [97.81, 100.71]	129 (95.56 %) [92.08, 99.03]	< 0.0001^a^ N vs. D: < 0.0001^a^ (0.286) N vs. K: 0.001^a^ (0.206) D vs. K: 0.120^a^ (N.C)
Grade 1	21 (15.56 %) [9.44, 21.67]	1 (0.74 %) [−0.71, 2.19]	6 (4.44 %) [0.97, 7.92]	
Grade 2	2 (1.48 %) [0.56, 3.52]	0 (0 %) [0.00, 0.00]	0 (0 %) [0.00, 0.00]	
**At 24 h**				
Grade 0	127 (94.07 %) [90.09, 98.06]	135 (100 %) [100.00, 100.00]	134 (99.26 %) [97.81, 100.71]	0.003^a^ N vs. D: 0.007^a^ (0.174) N vs. K: 0.036^a^ (0.145) D vs. K: 1^a^ (N.C.)
Grade 1	7 (5.19 %) [1.44, 8.93]	0 (0 %) [0.00, 0.00]	1 (0.74 %) [−0.71, 2.19]	
Grade 2	1 (0.74 %) [−0.71, 2.19]	0 (0 %) [0.00, 0.00]	0 (0 %) [0.00, 0.00]	

a Fisher's exact test.

b Chi-Square test. N.C., Not Computable Values are in n (%) and 95 % Confidence Interval (95 % CI) of percentage. Group N, D, and K: Group Normal saline, Dexamethasone, and Ketamine, respectively.

## Discussion

The present study demonstrated a significant reduction in the incidence of POST, postoperative coughing, and hoarseness of voice at various time intervals post-extubation among patients who underwent surgeries of duration 1 to 3 h and received intra-cuff inflation with Dexamethasone compared to intra-cuff use of Ketamine and normal saline. These findings suggest that Group D may offer the most favorable outcomes in mitigating postoperative sore throat, hoarseness, and cough at various intervals.

Endotracheal intubation ensures safety by safeguarding the airway; however, it increases susceptibility to POST. The proposed mechanisms involve an aseptic inflammatory response triggered by the irritation of the pharyngeal mucosa during laryngoscopy and persistent irritation of the tracheal mucosa caused by the presence of the endotracheal tube cuff. An additional significant factor is the potential for trauma to occur during intubation.^9^ Prevention of POST has been attempted through a variety of drugs and administration techniques.

Lipophilic medications permeate through the endotracheal tube cuff via diffusion. A small quantity of the drug traverses the cuff and has an anti-inflammatory effect on the mucosa. The cuff would serve as a reservoir for the medicines, facilitating diffusion. The suggested action of intra-cuff Dexamethasone and Ketamine likely relies on its anti-inflammatory properties, which include preventing leukocyte migration, preserving cell membrane integrity, and diminution of lysosomal release.^8^

Naqvi et al. conducted research including 70 patients, revealing that intra-cuff alkalinized lidocaine substantially reduced the intensity of POST, cough, hoarseness, and laryngeal spasm in the postoperative period compared to intra-cuff Ketamine.^10^ In a study of 80 patients, Bhat et al. compared the beneficial effects of Ketamine and alkalinized lidocaine injection in the endotracheal tube cuff to reduce POST in adult patients undergoing general anesthesia. They noted that alkalinized lidocaine was more effective than intra-cuff Ketamine.^11^ Similarly, in the present study, we noted that intra-cuff Dexamethasone was more effective than intra-cuff Ketamine in preventing POST. Dexamethasone permeates the cuff membrane and provides a prolonged local anti-inflammatory impact on the tracheal mucosa when delivered through the ETT cuff.^8^ This intervention markedly diminishes mucosal inflammation, post-extubation sore throat, and cough. Conversely, Ketamine predominantly acts as an N-Methyl-d-Aspartate (NMDA) receptor antagonist and has modest anti-inflammatory characteristics.^10^ Although Ketamine can alleviate airway irritation and inhibit reflex reactions like coughing, its effects are transient and mainly facilitated by local analgesia rather than significant anti-inflammatory actions. From a pharmacokinetic perspective, Dexamethasone's increased lipophilicity and extended duration of action confer a prolonged therapeutic window. Ketamine, while beneficial for immediate symptom alleviation, possesses a shorter duration of action and may be absorbed more rapidly. These combined mechanistic and pharmacokinetic benefits justify the preference for intra-cuff Dexamethasone over Ketamine in addressing post-extubation airway complications.

Rajan et al., in their study of 60 patients undergoing minor pelvic laparoscopic operations lasting less than two hours, discovered that intra-cuff Dexamethasone dramatically lowers the frequency and severity of POST, postoperative cough, and hoarseness of voice, which occur after general anesthesia with endotracheal intubation.^8^ Rafiei et al., in their study on 180 patients, found that using Dexamethasone to inflate the endotracheal tube cuff for mitigating post-extubation responses was as effective as lidocaine, although superior to normal saline.^12^ They considered it in clinical practice to enhance a patient's tolerance to anesthesia, particularly in cardiovascular illness, intracranial and intraocular hypertension, or pulmonary hyperreactivity. We discovered that intra-cuff Dexamethasone substantially decreased the incidence of POST and postoperative coughing and hoarseness at different time intervals after extubation.

Oliveira et al., in their study involving 154 children aged 4 to 12 years undergoing general anesthesia for elective tonsillectomy and adenotonsillectomy, discovered that intra-cuff alkalinized lidocaine, in conjunction with intravenous Dexamethasone, may effectively diminish sore throat 24 h postoperatively compared to air as the cuff insufflation medium.^6^ Magnesium, an N-methyl-d-aspartate receptor antagonist, possesses anti-nociceptive and anti-inflammatory effects. Singh et al. conducted a systematic review and meta-analysis of seven trials with 726 participants, revealing that the incidence of POST at 24 h was significantly reduced in the topical magnesium group (26 out of 363) compared to both the active and non-active control groups (89 out of 363); *p* = 0.00, RR = 0.22 (95 % CI 0.12‒0.39, I^2^ = 0 %).^13^

Reducing POST enhances the patient's tolerance to anesthesia, especially in cardiovascular disease, intracranial and intraocular hypertension, and pulmonary hyperreactivity. It minimizes the need for additional pain medication and may reduce hospital stays, enhancing the overall perioperative experience and quality of care. However, these factors were not measured in the current investigation. There can be potential side effects of intra-cuff medications, such as local tissue irritation or systemic absorption risks.^8^ However, we did not observe any adverse effects in the present study.

## Limitations

The present study has certain limitations. First, it was impossible to determine whether the reported throat pain was due to endotracheal intubation alone, as it may be associated with Ryle's tube position. However, the clinical benefit on the first postoperative day was noted either way. Furthermore, there may be inter-observer variability in assessing POST scores. Secondly, as the study was conducted in adult patients, some pain information was subjectively provided by patients. Patients may underestimate their sore throat pain without objective pain scales compared to surgical site pain. We could not assess the intra-cuff pressure due to fluid intrusion into the manometer, which might compromise the apparatus. No cuff pressure measurement device was used, which can introduce variability in drug diffusion and mucosal irritation. Further, pain related to airway management during intubation is directly related to cuff pressure, which can be a bias in this study.^14^ There was no control group (a placebo group with air in the cuff). Moreover, we did not measure any of the drug (Ketamine, Dexamethasone) serum concentrations. We also did not conduct long-term follow-up for the occurrence of laryngeal injuries or prolonged hoarseness beyond 24 h. Further studies are necessary to address these limitations in the future.

## Conclusion

Intra-cuff Dexamethasone appears to have the lowest incidence of postoperative sore throat, hoarseness, and cough at most time points during the early postoperative period, indicating its potential as an effective intervention for reducing postoperative discomfort.

## Declaration of competing interest

The authors declare no conflicts of interest.

## Appendix Supplementary materials

Image, application 1

## Footnotes

Supplementary material associated with this article can be found in the online version at doi:10.1016/j.bjane.2025.844651.
