# Supplementary material for: Comparison of the effect of intra-cuff normal saline, dexamethasone or ketamine for prevention of postoperative sore throat: a randomized controlled trial
Source: Braz J Anesthesiol. 2025 Jun 13;75(5):844651. doi: 10.1016/j.bjane.2025.844651 (PMC12369433; doi:10.1016/j.bjane.2025.844651)
Supplement: Supplementary file 1 [file mmc1.docx]

**BJAN-D-24-00652_ Supplementary Material**

**Supplementary Table 1** Absolute risk reductions and number needed to treat for post-operative sore throat in groups D and K at various time intervals.

| **Time** | **Group (n = 135 in each group)** | **ARR** | **NNT** |
| --- | --- | --- | --- |
| 2 hours | D | 0.252 | 4 |
|  | K | 0.067 | 15 |
| 6 hours | D | 0.222 | 5 |
|  | K | 0.025 | 40 |
| 12 hours | D | 0.178 | 6 |
|  | K | 0.078 | 13 |
| 24 hours | D | 0.059 | 17 |
|  | K | 0.044 | 23 |

Group D and K, Group Dexamethasone and Ketamine; ARR, Absolute Risk Reductions; NNT, Number Needed to Treat.

**Supplementary Table 2** Absolute risk reductions and number needed to treat for post-operative hoarseness in groups D and K at various time intervals.

| **Time** | **Group (n = 135 in each group)** | **ARR** | **NNT** |
| --- | --- | --- | --- |
| 2 hours | D | 0.156 | 7 |
|  | K | 0.052 | 20 |
| 6 hours | D | 0.163 | 7 |
|  | K | 0.081 | 13 |
| 12 hours | D | 0.096 | 11 |
|  | K | 0.044 | 23 |
| 24 hours | D | 0.037 | 27 |
|  | K | 0.015 | 67 |

Group D and K, Group Dexamethasone and Ketamine; ARR, Absolute Risk Reductions; NNT, Number Needed to Treat.

**Supplementary Table 3** Absolute risk reductions and number needed to treat for post-operative cough in groups D and K at various time intervals

| **Time** | **Group (n = 135 in each group)** | **ARR** | **NNT** |
| --- | --- | --- | --- |
| 2 hours | D | 0.170 | 6 |
|  | K | 0.085 | 12 |
| 6 hours | D | 0.237 | 5 |
|  | K | 0.081 | 13 |
| 12 hours | D | 0.163 | 7 |
|  | K | 0.037 | 28 |
| 24 hours | D | 0.059 | 17 |
|  | K | 0.015 | 67 |

Group D and K, Group Dexamethasone and Ketamine; ARR, Absolute Risk Reductions; NNT, Number Needed to Treat

**Supplementary File**

1. **Scoring for postoperative sore throat**

| **Severity** | **Grade** |
| --- | --- |
| No sore throat at any time since the operation | 0 |
| Minimal-Patient answered in the affirmative  when asked about a sore throat | 1 |
| Moderate-Patient complained of sore throat  on his/her own | 2 |
| The patient is in obvious distress | 3 |

1. **Scoring for postoperative hoarseness of voice**

| **Severity** | **Grade** |
| --- | --- |
| No complaint of hoarseness at any time  since the operation | 0 |
| Minimal-Minimal change in quality of speech. The patient answers in the affirmative only when enquired about | 1 |
| Moderate-Moderate change in the quality of speech of which the patient complains on his/her own | 2 |
| Severe-Gross change in the quality of voice  perceived by the observer | 3 |

1. **Scoring for postoperative cough**

| **Severity** | **Grade** |
| --- | --- |
| No cough at any time since the operation | 0 |
| Minimal | 1 |
| Moderate | 2 |
| Severe | 3 |
